# Testing for a Gap Junction-Mediated Bystander Effect in Retinitis Pigmentosa: Secondary Cone Death Is Not Altered by Deletion of Connexin36 from Cones

**DOI:** 10.1371/journal.pone.0057163

**Published:** 2013-02-27

**Authors:** Katharina Kranz, François Paquet-Durand, Reto Weiler, Ulrike Janssen-Bienhold, Karin Dedek

**Affiliations:** 1 Department of Neurobiology, University of Oldenburg, Oldenburg, Germany; 2 Institute for Ophthalmic Research, Centre for Ophthalmology, University of Tübingen, Tübingen, Germany; University of Florida, United States of America

## Abstract

Retinitis pigmentosa (RP) relates to a group of hereditary neurodegenerative diseases of the retina. On the cellular level, RP results in the primary death of rod photoreceptors, caused by rod-specific mutations, followed by a secondary degeneration of genetically normal cones. Different mechanisms may influence the spread of cell death from one photoreceptor type to the other. As one of these mechanisms a gap junction-mediated bystander effect was proposed, i.e., toxic molecules generated in dying rods and propagating through gap junctions induce the death of healthy cone photoreceptors. We investigated whether disruption of rod-cone coupling can prevent secondary cone death and reduce the spread of degeneration. We tested this hypothesis in two different mouse models for retinal degeneration (rhodopsin knockout and *rd1*) by crossbreeding them with connexin36-deficient mice as connexin36 represents the gap junction protein on the cone side and lack thereof most likely disrupts rod-cone coupling. Using immunohistochemistry, we compared the progress of cone degeneration between connexin36-deficient mouse mutants and their connexin36-expressing littermates at different ages and assessed the accompanied morphological changes during the onset (rhodopsin knockout) and later stages of secondary cone death (*rd1* mutants). Connexin36-deficient mouse mutants showed the same time course of cone degeneration and the same morphological changes in second order neurons as their connexin36-expressing littermates. Thus, our results indicate that disruption of connexin36-mediated rod-cone coupling does not stop, delay or spatially restrict secondary cone degeneration and suggest that the gap junction-mediated bystander effect does not contribute to the progression of RP.

## Introduction

Retinitis pigmentosa (RP) is a group of inherited retinal degenerative diseases characterized by a progressive loss of photoreceptor cells. RP is caused by mutations in a variety of genes (>40), predominantly expressed by rod photoreceptors [1]. Rod-specific mutations lead to primary cell death of rods, resulting in night blindness and tunnel vision in human patients. When the disease progresses, genetically normal cones also die, leading to loss of central vision and ultimately to blindness [1]. To date it is not understood how cell death propagates from dying rods to healthy cones in those forms of RP in which mutations occur only in rods [1]. However, studies from different RP mouse models showed that various mechanisms may contribute to secondary cone degeneration. Punzo et al. [2] suggested that cones may starve to death because progressive rod loss disrupts the physical interaction between photoreceptors and the supporting retinal pigment epithelium, thereby depriving cones from nutrients [2], [3]. Also, deprivation from a rod-derived cone viability factor, which may be constantly released by healthy rod photoreceptors, could result in secondary cone death [4], [5]. Other studies suggested that cell death-inducing molecules, potentially released into the extracellular space by activated microglia cells [6], [7], were involved. The *gap junction-mediated bystander effect* provides another explanation that is often considered [8], [9]. In this scenario, the cell death-inducing signal is not released into the extracellular space but permeates from dying rods through gap junctions directly to healthy cones, thereby carrying cell death-promoting signals from one photoreceptor type to the other [8]. This hypothesis is supported by studies demonstrating that gap junction channels, which allow passage of small molecules (below ∼1 kDa), are involved in controlling the death of retinal cells during development and after traumatic injury. Dying neuroblasts, for instance, generate gap junction-permeant apoptotic signals that mediate bystander killing during retinal development [9]. Studies on a trauma model in chicken retina demonstrated the spread of apoptotic cell death through gap junctions after mechanical damage [10].

To the best of our knowledge, to date the potential contribution of a gap junction-mediated bystander effect (mediated by rod-cone coupling) to secondary cone degeneration in RP has never been investigated. Therefore, we crossbred two different mouse models for RP with mice deficient for the gap junction protein connexin36 (Cx36). As Cx36 is expressed on the cone side of the gap junction [11]–[13], deletion of this connexin leads to a disruption of Cx36-dependent rod-cone coupling [14]–[16]. To investigate the influence of photoreceptor coupling on different stages of cone degeneration, we chose two mouse models for RP with different time courses of photoreceptor degeneration: the rhodopsin knockout (*Rho* ^−/−^) mouse is a slow model of photoreceptor degeneration [17]. The long time period between the onset of cone degeneration and the actual death of cones makes this RP mouse model suitable to investigate the influence of photoreceptor coupling on early events in cone degeneration. Later stages of cone degeneration, when the majority of cones have died, were analyzed in the *rd1* mouse, which represents a well-established model of fast photoreceptor degeneration [18].

## Results

### Cx36 Expression is not Altered in Rho^−/−^ and rd1 mice

**Figure 1:**
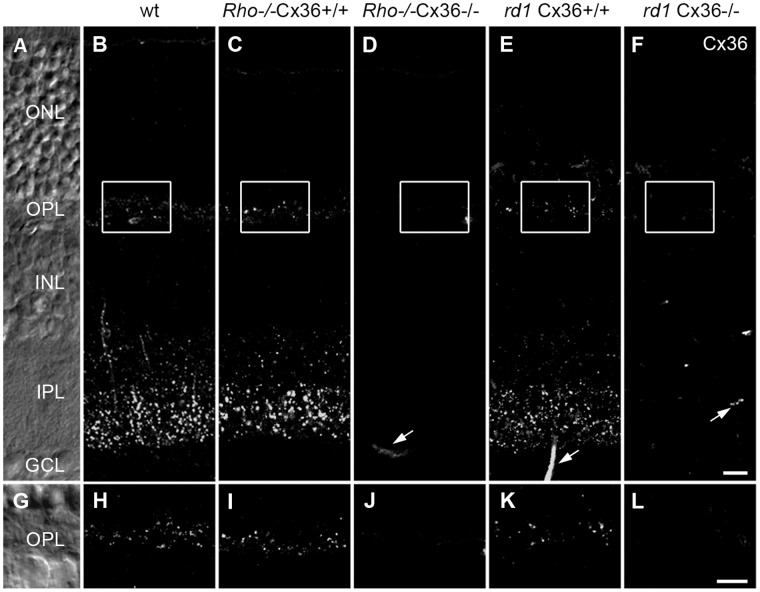
Distribution of Cx36 in retinal degeneration mouse models. Cx36 antibody staining in vertical sections of wild-type (wt) mice (B), *Rho* ^−/−^ (pw5) (C) and *rd1* mice (p21) (E) depicted the typical distribution pattern of Cx36 in the OPL and in the IPL. The staining is absent in *Rho* ^−/−^ and *rd1* mice with a targeted deletion of Cx36 (D, F). Cx36 immunoreactivity in magnified regions in the OPL of wt (H), *Rho* ^−/−^Cx36^+/+^ (I) and *rd1*Cx36^+/+^ mice (K) produced fine punctuate labeling in the outer plexiform layer (OPL) where rods and cones are electrically coupled by Cx36. This staining was absent in the OPL of Cx36-deficient *Rho* ^−/−^ (J) and *rd1* mutants (L). Residual staining (arrows) was caused by unspecific binding of the secondary antibody to blood vessels and was also present in controls (stainings without primary antibody). Retinal layers are indicated in the Nomarski micrographs (A, G). Scale bars = 10 µm in F (applies to A–F); in L (applies to G–L).

Physiological and structural analysis in wild-type (wt) retinas previously demonstrated that cone photoreceptors are functionally coupled to rods [14], [15], [19]–[22]. This coupling is mediated by the gap junction protein connexin36 (Cx36) expressed on the cone side [11], [12], [14] and another, yet unknown connexin on the rod side. To examine if secondary cone degeneration in *Rho* ^−/−^ and *rd1* mice may potentially be influenced by the deletion of the cone connexin, we first investigated if both degeneration models exhibit a normal distribution of Cx36 in the outer plexiform layer (OPL; Fig. 1). Figure 1B shows the characteristic punctate distribution of Cx36 in vertical wt sections. Consistent with previous studies [12], Cx36 immunoreactivity is stronger in the inner plexiform layer (IPL) than in the OPL where it is attributed to the dendrites of OFF bipolar cells and to cone photoreceptor endings [12]. A similar Cx36 distribution was obtained in retina sections from *Rho* ^−/−^ [postnatal week (pw) 5, Fig. 1C] and *rd1* mice [postnatal day (p) 21, Fig. 1E]. Higher magnification revealed that the overall density of Cx36-positive puncta in the OPL of *Rho* ^−/−^ (Fig. 1I) and *rd1* mice (Fig. 1K) was comparable to wild type (Fig. 1H), suggesting that Cx36 expression and most likely also rod-cone coupling are not altered in *Rho* ^−/−^ and *rd1* mice.

To disrupt rod-cone coupling, both models were crossbred with Cx36 knockout mice (Cx36^−/−^) [23]. As expected, Cx36 immunosignals were absent in retinas from *Rho* ^−/−^Cx36^−/−^ (Fig. 1D, J) and *rd1*Cx36^−/−^ (Fig. 1F, L) mice, allowing to directly test the effect of Cx36-dependent photoreceptor coupling on secondary cone degeneration in RP.

### Time Course of Photoreceptor Degeneration

**Figure 2:**
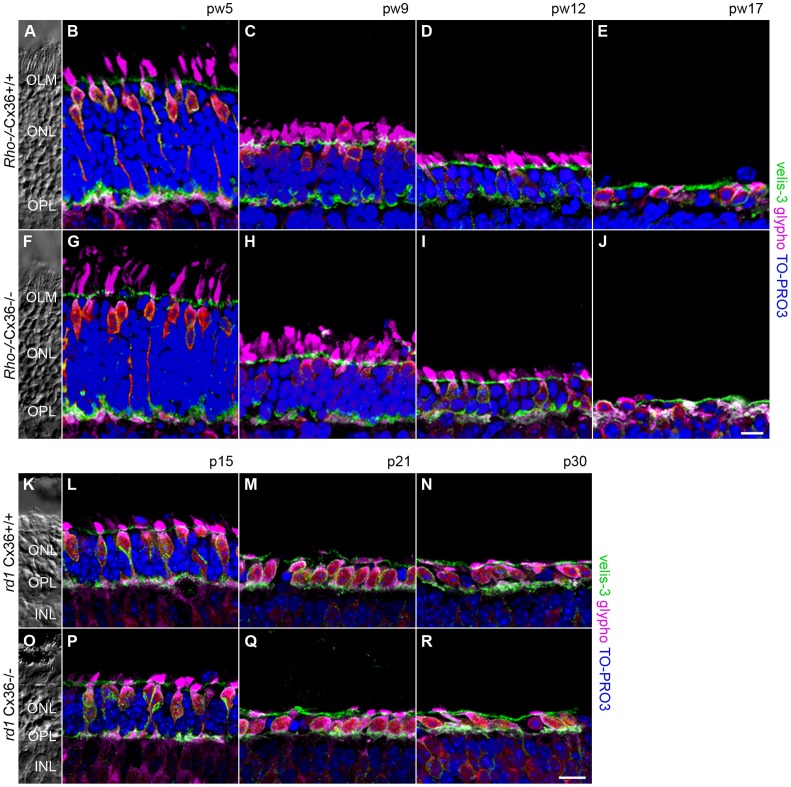
Progression of photoreceptor degeneration in *Rho* ^−/−^ and *rd1* mutants. Projections of collapsed confocal scans showed the outer retinal morphology in vertical retina slices of Cx36-expressing (A–E, K–N) and Cx36-deficient (F–J, O–R) *Rho* ^−/−^ (A–J) and *rd1* mice (K–R) at different developmental stages. Labeling of cone photoreceptors for glypho (magenta) and staining of photoreceptor terminals and the OLM for velis-3 (green) illustrated the progress of photoreceptor degeneration. Nuclei were stained with TO-PRO-3 (blue). Retinal layers are indicated on transmission photographs in A, F, K, O. Scale bar = 10 µm in J (applies to A–J); in R (applies to K–R).

To investigate if deletion of the cone connexin alters the progression of secondary cone degeneration, we compared the outer retinal morphology of Cx36-expressing (Fig. 2A–E; K–N) and Cx36-deficient (Fig. 2F–J; O–R) *Rho* ^−/−^ (Fig. 2A–J) and *rd1* mice (Fig. 2K–R). Vertical cryosections were counterstained with antibodies against glycogen phosphorylase (glypho), to label the entire cone photoreceptor [24], and antibodies against velis-3, to stain the outer limiting membrane (OLM) and photoreceptor terminals [25]. Retinal layering was visualized with the nucleic acid stain TO-PRO-3. Figure 2 shows the difference in time course of rod photoreceptor degeneration between both models: the slow degeneration over a time period of four months in *Rho* ^−/−^Cx36^+/+^ and *Rho* ^−/−^Cx36^−/−^ mice (Fig. 2A–J) and the fast degeneration within one month in *rd1*Cx36^+/+^ and *rd1*Cx36^−/−^ mice (Fig. 2L–R).

In *Rho* ^−/−^ mice, previous studies showed that photoreceptor degeneration starts with the loss of rods around pw3 to pw4 [17]. Consistently, at pw5 the ONL of *Rho* ^−/−^ mice became thinner as the number of nuclei was reduced (Fig. 2B; 10–12 rows of nuclei) compared to wt mice (12–14 rows of nuclei; not shown). At this age, cones, however, were not affected by the primary degeneration and displayed their normal shape (Fig. 2B). Secondary cone degeneration starts around pw6 [26] when cones begin to change their morphology. With progressive rod loss, indicated by a further thinning of the ONL, cones became shorter (Fig. 2C) and gradually lost their outer (Fig. 2C) and inner segments (Fig. 2D, E). However, the major phase of cone death did not begin before pw17 [2]. At this time-point, most rods had died and the ONL was reduced to a single row of nuclei (Fig. 2E); velis-3-labeled photoreceptor terminals were hardly discernible (Fig. 2E). However, similar changes in the outer retinal morphology were also observed in *Rho* ^−/−^Cx36^−/−^ littermates (Fig. 2F–J).

In contrast to rhodopsin-deficient mice, photoreceptor degeneration in the *rd1* mouse model occurred within days and not weeks (Fig. 1K–R). Primary rod degeneration started during retinal development around p11 [2] and reduced the ONL at p15 to half of its width (Fig. 2L). Cone morphology was already impaired in p15 *rd1*Cx36^+/+^ mice (Fig. 2L) as cones became smaller in size and the inner and outer segments regressed (Fig. 2L–N). In line with previous studies [2], our results showed that rod degeneration in *rd1*Cx36^+/+^ mice progressed rapidly and left only one row of nuclei in the ONL after the major phase of rod death at p21 (Fig. 2M). Around this time point, secondary cone death is initiated [27]. Consistently, the number of cells in the ONL further decreased (Fig. 2N) and cones lost nearly all processes and their characteristic shape (Fig. 2M, N). From p21 on, glypho-positive immunoreactivity around remaining nuclei in the ONL indicated that the majority of remaining cells represented cones. When we analyzed the time course of cone degeneration in *rd1* mice lacking Cx36 (*rd1*Cx36^−/−^), we did not find any differences from *rd1* mice (Fig. 2P–R).

### Remodeling of Second Order Neurons

In mouse models for RP, photoreceptor degeneration is accompanied by morphological changes of downstream neurons, which respond to the loss of glutamatergic input from photoreceptors with structural reorganization. In the *Rho* ^−/−^ and *rd1* mouse models, horizontal cells (HC) as well as some ON and OFF bipolar cell types strongly reorganize [28]–[31]. To determine whether Cx36 deficiency changes retinal remodeling, we compared the morphologies of HC and distinct bipolar cell types between Cx36-expressing and Cx36-deficient *Rho* ^−/−^ and *rd1* mice.

**Figure 3:**
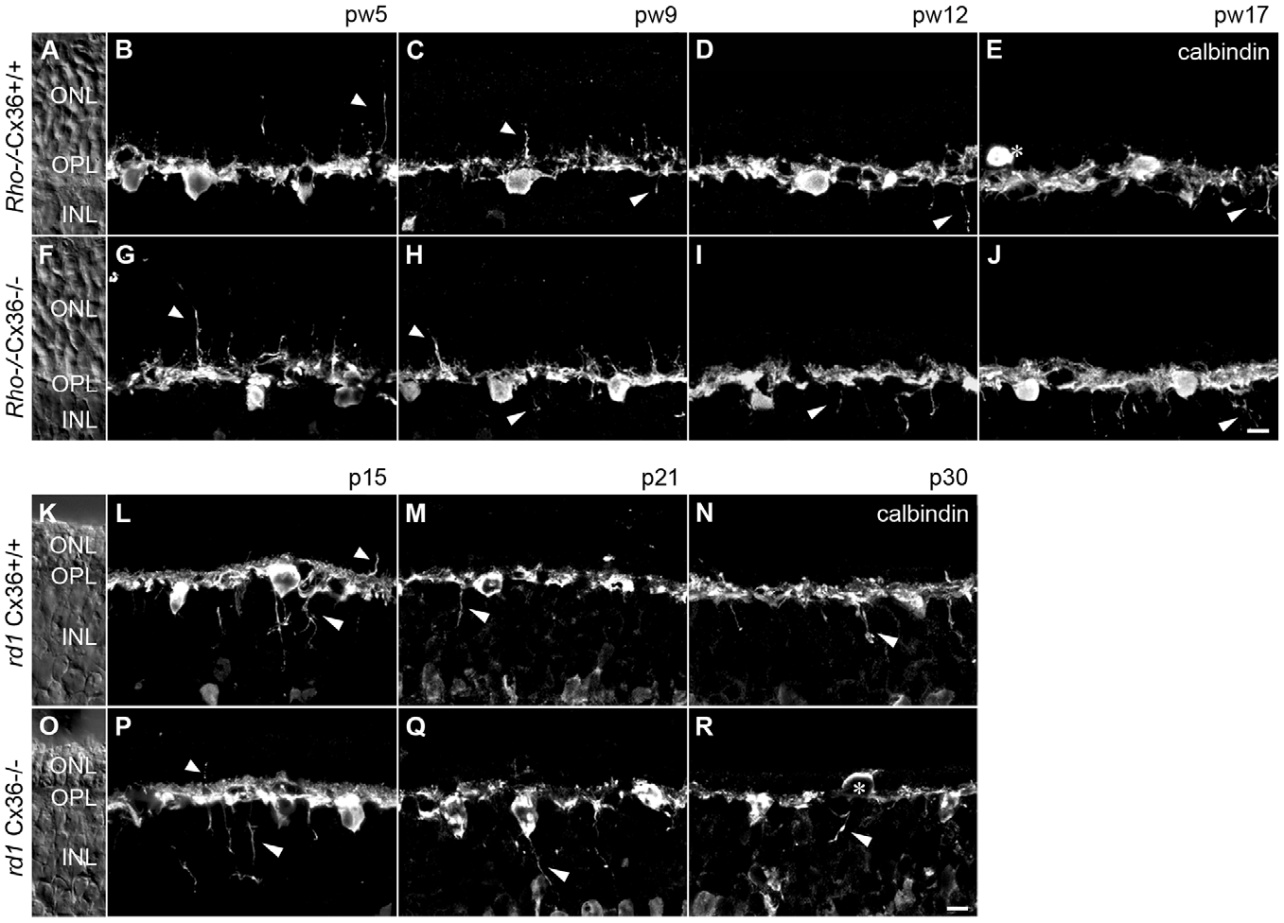
Reorganization of horizontal cells is not changed by Cx36 deficiency. Horizontal cells were labeled with anti-calbindin antibodies at various ages in *Rho* ^−/−^Cx36^+/+^ (A–E), *Rho* ^−/−^Cx36^−/−^ (F–J) and *rd1*Cx36^+/+^ (K–N), *rd1*Cx36^−/−^ mice (O–R). Independent of Cx36 deletion, the reorganization of HC was initiated by the sprouting of processes into the ONL (B, C, G, H, L, P; short arrowhead). However, at later stages, these processes retracted and horizontal cell dendrites reached progressively into the INL (C–E, H–J, L–N, P–R; long arrowhead). Horizontal cell somata were occasionally found displaced to the ONL (E, R, asterisks). Nomarski micrographs (A, F, K, O) indicate the retinal layering. Scale bar = 10 µm in J (applies to A–J); in R (applies to K–R).

HC were immunolabeled with antibodies against the calcium-binding protein calbindin [32] (Fig. 3). Consistent with previous studies [28], HC staining in *Rho* ^−/−^Cx36^+/+^ retinas revealed an initial outgrowth of processes into the ONL up to pw9 (Fig. 3B, C; short arrowhead). In older animals (pw12, pw17) HC sprouts retracted from the ONL and progressively ramified into the inner nuclear layer (INL) (Fig. 3D, E; long arrowhead). At pw17, HC somata occasionally switched their position from the distal INL into the ONL (Fig. 3E; asterisk). No differences in the rearrangement of HC processes and somata were observed between Cx36-expressing and Cx36-deficient siblings (Fig. 3F–J).

HC reorganization showed similar hallmarks in *rd1* mice and *rd1* mice lacking Cx36, but it occurred faster than in *Rho* ^−/−^ mutants. Already at p15, HC processes, mostly originating from axonal complexes [30], protruded into the INL (Fig. 3L, long arrowhead). At this time point, some smaller sprouts were still present in the ONL (Fig. 3P, short arrowhead). While HC extensions in the ONL retracted after p21, the long processes in the INL persisted over the investigated period of time (Fig. 3M, N). At p30, HC somata were occasionally displaced to the ONL (Fig. 3R, asterisk). Thus, deletion of Cx36 from the rod-cone gap junction did not change the reorganization of HC in *rd1* mice, which followed a similar time course as reported in other studies [29]–[31].

**Figure 4:**
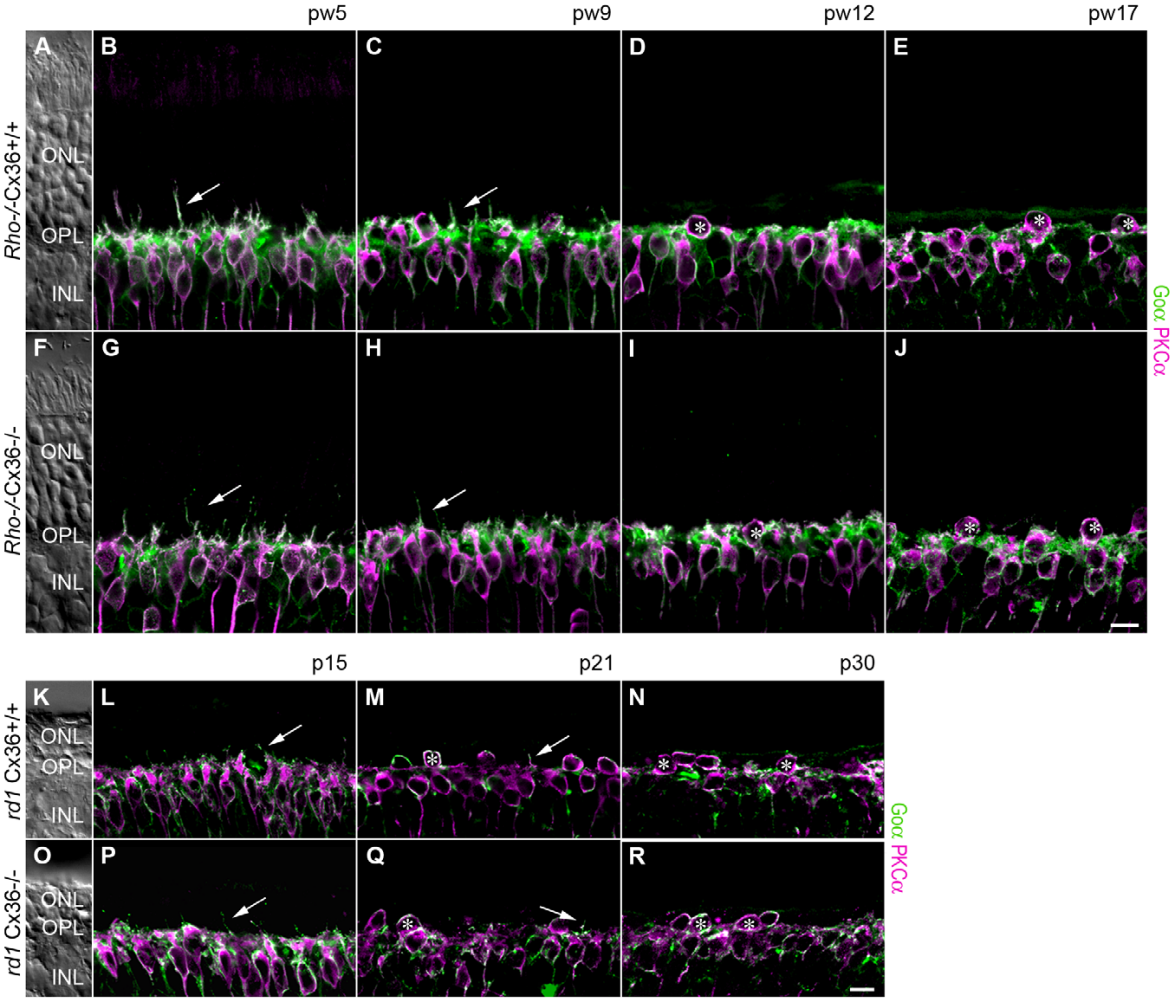
Deletion of Cx36 did not affect remodeling of ON bipolar cells in *Rho* ^−/−^ and *rd1* mice. Vertical sections of the retina were double-stained for G0α (green), a marker for all ON BC, and PKCα (magenta), a marker for rod BC. In Cx36-expressing *Rho* ^−/−^ and *rd1* mutants, rod bipolar cell dendrites sprouted into the ONL (B, C, L, M; long arrow) at the onset of degeneration. With progressing photoreceptor degeneration, all ON bipolar cell dendrites were retracted (D, M). In both disease models, PKCα-positive cell somata were frequently found displaced to the ONL (D, E, M, N, asterisks). Remodeling was similar in Cx36-deficient *Rho* ^−/−^ (G–J) and *rd1* littermates (P–R). Nomarski micrographs (A, F, K, O) indicate the retinal layering. Scale bars = 10 µm in J (applies to A–J), in R (applies to K–R).

To investigate the remodeling of bipolar cells, we immunolabeled the ON bipolar cell (BC) population with antibodies against the G-protein subunit G_0α_ [33]. To distinguish rod from cone ON BC, rod BC were additionally stained with antibodies directed against PKCα [32] (Fig. 4). During photoreceptor degeneration, BC reorganized with distinct morphological characteristics which were similar in both, *Rho* ^−/−^Cx36^+/+^ and *Rho* ^−/−^Cx36^−/−^ mice. At pw5, the dendritic organization appeared normal, except for some PKCα-positive rod BC dendrites sprouting into the ONL (Fig. 4B, C, G, H; long arrow). The dendrites of PKCα-negative cone ON BC, in contrast, did not extend into the ONL. While photoreceptor degeneration progressed, almost all ON BC dendrites were retracted and were almost completely absent at pw12 (Fig. 4D). From pw12 onward, rod BC often switched their position into the ONL (Fig. 4D, E, I, J; asterisk).

Similar changes were observed in *rd1*Cx36^+/+^ and *rd1*Cx36^−/−^ mutants, in agreement with previous findings in *rd1* mice [29]–[31]: ON BC dendrites were only rudimentarily developed at the early age of p15. Occasionally, at p15 and p21, small rod BC dendrites sprouted into the ONL, presumably due to the impaired synaptic transmission from dying rods (Fig. 4L, P, long arrow). The dendrites of G_0α_-labeled rod and cone ON BC progressively retracted and were completely absent after 21 days of age (Fig. 4M, N). Similar to the older *Rho* ^−/−^ mutants, an increasing number of rod BC somata was displaced to the ONL (Fig. 4 N, R; asterisk).

**Figure 5:**
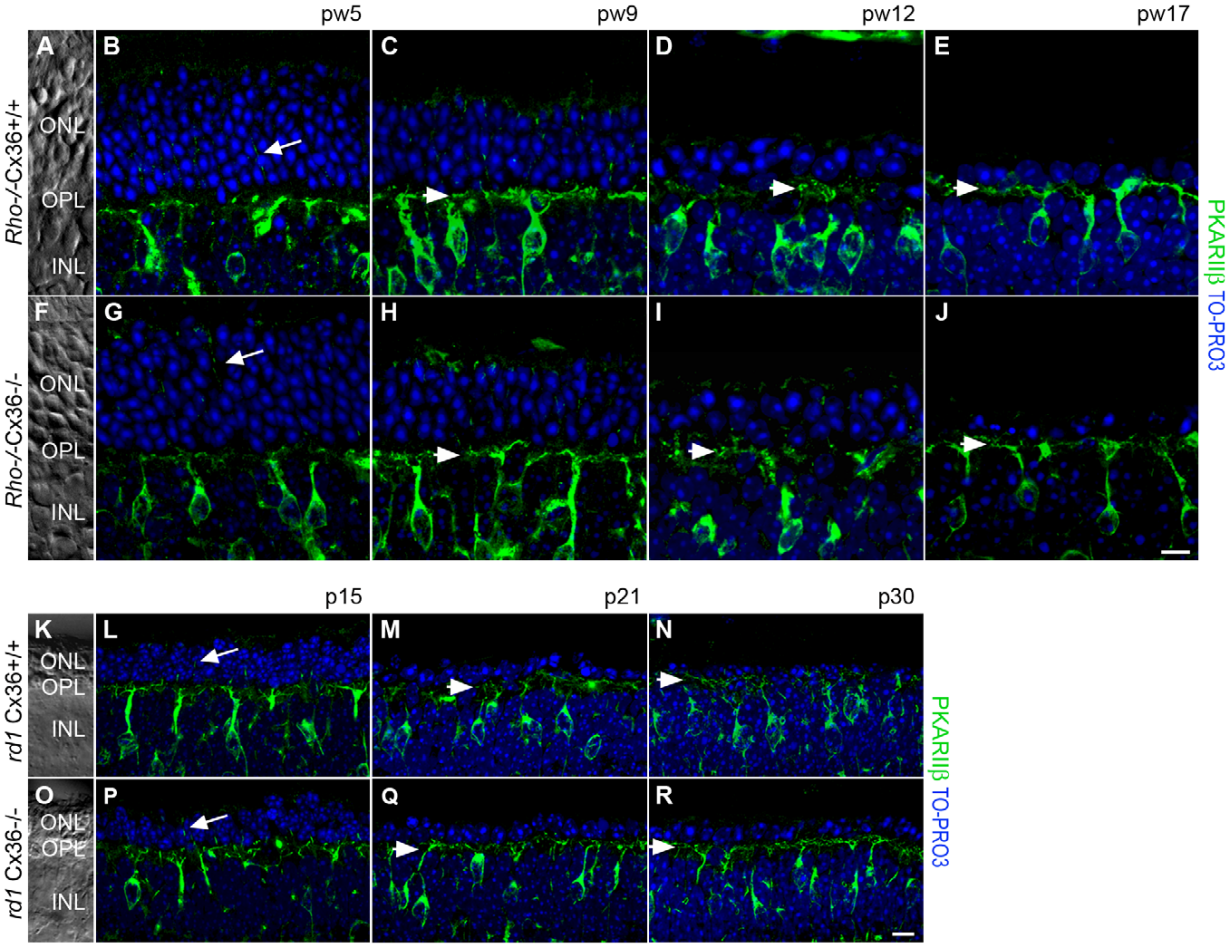
Deletion of Cx36 did not affect remodeling of type 3b OFF bipolar cells. Morphological changes of type 3b OFF cone bipolar cells visualized with anti-PKARIIβ antibodies (green). Nuclei were counterstained with the nucleic acid stain TO-PRO-3 (blue). During photoreceptor degeneration, dendrites of type 3b BC developed very fine processes protruding through the ONL up to the OLM (arrows). When the ONL became thinner with disease progress, these dendrites retracted. Additional dendrites persist in the OPL of all tested ages (short arrow). Morphological changes did not differ between Cx36-expressing and Cx36-deficient *Rho* ^−/−^ (A–J) and *rd1* mice (K–R). Scale bars = 10 µm in J (applies to A–J), in R (applies to K–R).

For OFF bipolar cells, we used the rod- and cone-contacting type 3b BC as an example because type 3b cells were found to reorganize when transmission from photoreceptors is impaired [34], [35]. Type 3b cells were specifically labeled with antibodies against PKARIIβ [36]. In both RP models, this cell type responded similarly to photoreceptor degeneration. Type 3b cells developed very fine processes, which extended through the entire ONL up to the OLM, presumably reaching out for photoreceptor input [35]. These changes were already detected at pw5 in *Rho* ^−/−^Cx36^+/+^ and p15 in *rd1* Cx36^+/+^ (Fig. 5B, L; long arrows), respectively. Sprouted dendrites, however, were almost completely retracted with progressive thinning of the ONL while other dendrites remained in the OPL even at later degeneration stages (Fig. 5C–E, M, N, short arrows). Remodeling was similar in the respective Cx36-deficient *Rho* ^−/−^ and *rd1* littermates (Fig. 5F–J, O–R).

Thus, our immunostainings clearly demonstrated that photoreceptor degeneration resulted in massive morphological changes of second order neurons. However, reorganization during photoreceptor degeneration in *Rho* ^−/−^ and in *rd1* mice was unaffected by the lack of Cx36; deletion of the cone connexin did not change the extent or the time course of retinal reorganization in RP mouse models.

### Loss of Cone Outer Segments at the Onset of Secondary Cone Degeneration

To extend our investigations with more sensitive and direct measurements, we examined the effect of Cx36 deficiency on the onset (in *Rho* ^−/−^ mice) and during later stages (in *rd1* mice) of secondary cone degeneration.

**Figure 6:**
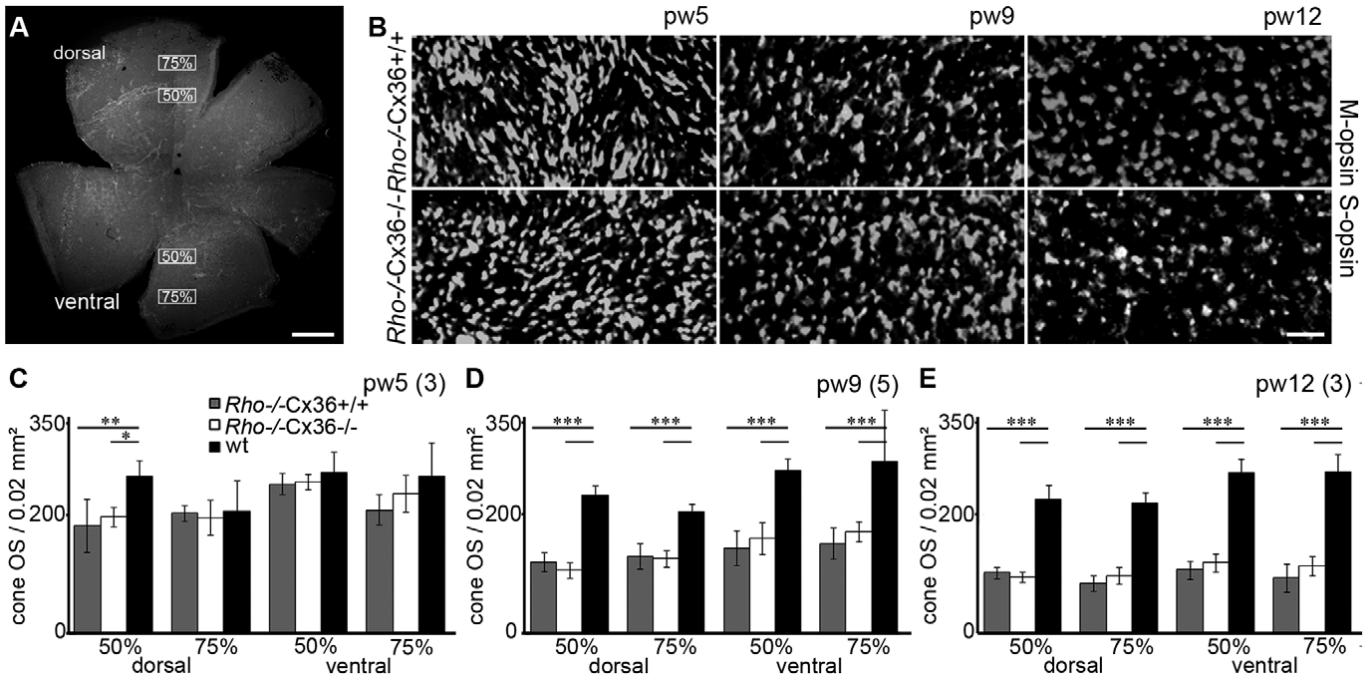
Cx36 deletion did not affect the loss of cone outer segments in *Rho* ^−/−^ mice. Onset of cone degeneration was assessed by the quantification of cone outer segments (COS) in the central and peripheral part in retinal whole-mounts (in a distance of 50% and 75% dorsal and ventral of the optic nerve, as indicated in A). COS were labeled with antibodies against M- and S-opsin. Representative examples of quantified regions in the dorsal part of the retina (75%) in 5-, 9- and 12-week-old *Rho* ^−/−^Cx36^+/+^ and *Rho* ^−/−^Cx36^−/−^ mice are shown in B. The number of COS per 0.02 mm^2^ was quantified in Cx36-expressing (grey) and Cx36-deficient (white) *Rho* ^−/−^ mutants and same-aged wild-type controls (black) at the age of pw5 (C), pw9 (D) and pw12 (E). Values are given as mean ± SD; a t-test was used to compare means: *, p<0.05; **, p<0.01; ***, p<0.001. n specifies the number of animals. Scale bars = 300 µm in A; 25 µm in B.

During the process of photoreceptor degeneration, cells pass through different metabolic changes. One very early event indicating the onset of cone degeneration is the retraction and subsequent loss of cone outer segments (COS) [2]. In *Rho* ^−/−^ mutants, it takes more than 10 weeks from the retraction of COS until cones get lost (Fig. 2A–J). Therefore, this RP model is well suited to investigate the influence of Cx36-dependent rod-cone coupling on the onset of cone degeneration. We directly analyzed this by comparing the progressive loss of COS between 5-, 9- and 12-week-old *Rho* ^−/−^Cx36^+/+^, *Rho* ^−/−/^Cx36^−/−^, and wt animals (Fig. 6). COS were stained in retinal whole-mounts with antibodies against both cone opsins (S-opsin, M-opsin) [37]. The number of stained COS was quantified in four different regions of interest (ROI; 100×200 µm^2^), 50% and 75% along the dorsal-ventral axis (with 100% corresponding to the distance between the optic nerve head and the retina edge) to control for eccentricity-related differences (Fig. 6A). Representative ROI examples demonstrate the progressive loss of COS from 5- to 12-week-old *Rho* ^−/−^Cx36^+/+^ and *Rho* ^−/−^Cx36^−/−^ animals (Fig. 6B). However, as these examples show no obvious differences in the density of COS between same-aged Cx36-expressing and Cx36-deficient *Rho* ^−/−^ mutants (Fig. 6B), we quantified the number of COS in retinas from at least three different animals for each genotype (Fig. 6C–E). At pw5, there were no significant differences in the number of COS between *Rho* ^−/−^Cx36^+/+^, *Rho* ^−/−^Cx36^−/−^ and wt controls (Fig. 6C; p>0.0803 for all comparisons, n = 3), except for the central part of the dorsal retina. In this region, COS were significantly reduced in both transgenic animals, when compared to wt mice (p = 0.0178, *Rho* ^−/−^Cx36^+/+^, wt; p = 0.0093, *Rho* ^−/−^Cx36^−/−^, wt, n = 3). As cone degeneration starts in the central retina in *Rho* ^−/−^ mice [2], we assume that this decrease might represent the onset of cone degeneration. While the number of COS remained almost constant in wt controls, a substantial fraction of COS was lost in 9- and 12-week-old *Rho* ^−/−^Cx36^+/+^ and *Rho* ^−/−^Cx36^−/−^ mice, indicating that early cone degeneration now covered a large retinal area (Fig. 6D, E). However, because the density of COS showed no significant differences between these mutants (p>0.1089 for all comparisons; n = 5, pw9, n = 3, pw12), we conclude that the loss of COS is not delayed or prevented by the deletion of the cone connexin from the rod-cone gap junction (Fig. 6C–E).

### Secondary Cell Death and Loss of Cone Photoreceptors at a Late Disease Stage

**Figure 7:**
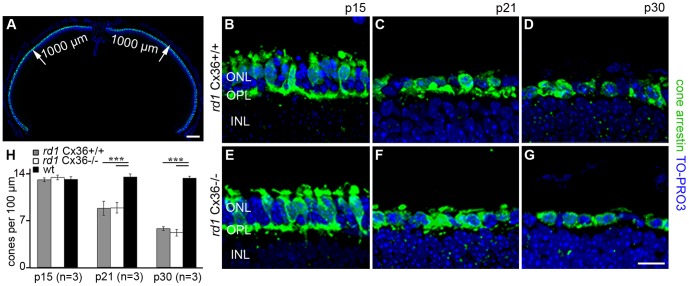
Time course of cone loss in *rd1*Cx36^+/+^ and *rd1*Cx36^−/−^ mice. Quantification of cones in vertical sections of the central retina (defined up to a distance of 1,000 µm from the optic nerve), as indicated in A. B–G show magnifications of the quantified regions in the central ONL of cone arrestin-labeled vertical sections from *rd1*Cx36^+/+^ (B–D) and *rd1*Cx36^−/−^ (E–G) mice at different ages. The bar graph (H) displays the quantification of cone arrestin-positive cells per 100 µm length at different ages in *rd1*Cx36^+/+^ (grey), *rd1*Cx36^−/−^ (white) and wt mice (black). The number of cone photoreceptors in both transgenic mouse lines was reduced at p21 and p30 compared to same-aged wt controls. There are no statistical differences between Cx36-expressing and Cx36-deficient *rd1* mice. Values are given as mean ± SD; a t-test was used to compare means: ***, p<0.001. n specifies the number of animals. Scale bars = 200 µm in A; 10 µm in G (applies to B–G).

**Figure 8:**
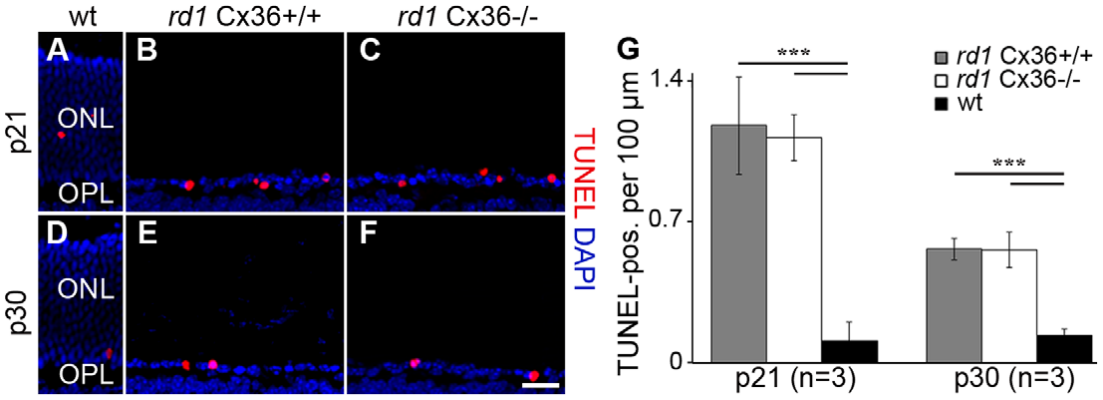
Cell death rate in *rd1*Cx36^+/+^, *rd1*Cx36^−/−^ and wt mice. Dying cone photoreceptors in vertical sections of wild-type (wt) (A, D), *rd1*Cx36^+/+^ (B, E), *rd1*Cx36^−/−^ mouse retina (C, F) at the ages of p21 (A–C) and p30 (D–F) were labeled by TUNEL staining (red); nuclei were stained with DAPI (blue). Sections from wt controls indicate the normal retinal layering (A, D). The bar graph (G) summarizes the quantification of TUNEL-positive cells in the ONL in *rd1*Cx36^+/+^ (grey), *rd1*Cx36^−/−^ (white) and wt mice (black). The amount of TUNEL-positive cells in both *rd1* genotypes was significantly increased when compared to wt mice. Comparisons between same-aged *rd1* mutants revealed no statistical differences. Values are given as mean ± SD; a t-test was used to compare means: ***, p<0.001. n specifies the number of animals. Scale bars = 20 µm in F (applies to A–F).

The fast progression of photoreceptor degeneration in *rd1*Cx36^+/+^ and *rd1*Cx36^−/−^ caused an early and rapid retraction of COS [38] as indicated in Figure 2K–R. Thus, the *rd1* mouse model is not suitable for analyzing the loss of COS over time. Instead, we used the *rd1* model to directly examine the influence of Cx36 deletion on cone loss (Fig. 7) and cell death (Fig. 8). We measured cone loss by quantifying remaining cones in vertical retina sections labeled with antibodies against cone arrestin (Fig. 7A). As the retinal degeneration in *rd1* mice followed a center-to-periphery gradient [18], cones were quantified only in the central part of the retina, up to a distance of 1,000 µm from the optic nerve (Fig. 7A). Cone arrestin labeling revealed the ongoing deformation and loss of cones from p15 to p30 (Fig. 7B–G). There were no obvious differences in shape or number of cones between same-aged Cx36-expressing (Fig. 7B–D) and Cx36-deficient *rd1* mice (Fig. 7E–G). These observations were confirmed by quantitative data (Fig. 7H). The number of cones was the same in *rd1* mutants and wt controls at p15, indicating that the loss of cones was not yet initiated at this age. In contrast, the number of cones decreased by one third in *rd1*Cx36^+/+^ and *rd1*Cx36^−/−^ mice from p15 to p21 (p = 7.1×10^−7^, n = 3) and by almost another third from p21 to p30 (p = 0.5×10^−5^, n = 3), confirming the progressive loss of cones during this period [2], [27]. However, there was no statistical difference in the number of cone photoreceptors between age-matched Cx36-expressing and Cx36-deficient *rd1* mutants (p15: p = 0.2993; p21: p = 0.9271; p30: p = 0.1386).

We used a second approach to analyze the influence of Cx36 deletion on the late stage of cone degeneration. We compared the number of dying cells between Cx36-expressing and Cx36-deficient *rd1* siblings (Fig. 8). DNA fragmentation represents a late event in cell death. The emerging DNA nick ends were detected *in situ* with TUNEL staining. TUNEL-positive cells were quantified in the central retina of *rd1* Cx36^+/+^, *rd1* Cx36^−/−^ and wt mice at the ages of p21 and p30, i.e., at times when secondary cone loss progressed [2], [27], as shown in Figure 7. Because most rods have died at p21 [18] and the majority of remaining cells in the ONL represent cone photoreceptors (Fig. 2M, N, Q, R), TUNEL-positive nuclei in the ONL most likely represent dying cones (Fig. 8B, C, E, F). As expected, comparing the number of dying cells between same-aged *rd1* mutants and wt mice revealed a significantly increased number of TUNEL-positive cells in both *rd1* mutants when compared to wt mice (p<0.0004 for all comparisons), with a higher number of TUNEL-positive cells at p21 than at p30 (Fig. 8G; *rd1*Cx36^+/+^: p = 0.0020; *rd1*Cx36^−/−^: p = 0.0032). However, deletion of Cx36 did not change the time course or the extent of secondary cone death as it did not affect the number of dying cones (Fig. 8G; p21: p = 0.5231; p30: p = 0.9296; n = 3).

## Discussion

The bystander effect is an established mechanism to explain the propagation of cell death from dying cells to healthy neighbors and was demonstrated in various *in vitro* [39], [40] and *in vivo* systems [41]–[43]. To explain the secondary death of genetically normal cones in RP, Ripps [8] proposed a gap junction-mediated bystander effect and suggested that gap junctions provide an avenue by which toxic intermediates generated by dying rods are transmitted to healthy cones [8], [9]. We tested this hypothesis in two different mouse models for RP, the *rd1* and the *Rho* ^−/−^ mouse, by targeted deletion of the gene coding for Cx36, the gap junction-forming protein on the cone side. We assessed the progress of the degenerative disease at various levels; however, we did not find any differences in secondary cone degeneration between RP mice with and without Cx36.

There are several possibilities why no effect on secondary cone degeneration was found and therefore we will discuss in the following the impact of Cx36, the parameters assessed, and the gap junction-mediated bystander effect in RP.

### Cx36 Expression in RP Mouse Models

Electrical coupling between rod and cone photoreceptors is the basic premise of the bystander hypothesis and has been demonstrated in a variety of animal models [44], including the mouse [14], [15], [19], [22], [45]. Although for the mouse only the connexin on the cone side is known (Cx36) [12], [13], several studies show that disruption of Cx36 is sufficient to disrupt rod-cone coupling [14]–[16]. If Cx36 plays a role in secondary cone death, mouse models for RP should express Cx36 in the OPL. This was indeed the case; we found that Cx36 expression was similar to wild-type controls at early (*Rho* ^−/−^, pw5) and later RP stages (*rd1*, p21), confirming that Cx36-containing gap junctions may indeed mediate bystander killing. Breeding *Rho* ^−/−^ and *rd1* mice with Cx36^−/−^ mice abolished the expression of Cx36 (Fig. 1) and allowed a test of the bystander hypothesis.

### Assessing Secondary Cone Degeneration

The impact of Cx36 deletion on secondary cone degeneration was assessed by evaluating the rearrangement of second order neurons, the loss of COS as an indication for the *onset* of secondary cone degeneration, and the numbers of dying and residual cones as an indication for the *progress* of secondary cone death.

The slow time course of retinal degeneration in rhodopsin-deficient mice was similar as in previous studies [2], [17]. Rods do not form outer segments and die over a period of 17 weeks. Cones develop normally at first, leading to a supernormal response in electroretinograms around pw5 [26], but start to degenerate from pw6 to pw13 [26], loosing inner and outer segments until pw17 (Fig. 2E). In contrast to *Rho* ^−/−^ mice, degeneration in *rd1* mice was much faster: rods start to degenerate around p10–11 and are almost completely lost until p20 (Fig. 2N) [38], [46]. Cone photoreceptors, although unaffected by the *Pde6β* mutation, start to degenerate shortly after rod degeneration begins [27]. Although most cones die within 20–30 days, cone cell death may take up to six months to be completed [2], [27]. Thus, the time points investigated were suitable to analyze the onset (*Rho* ^−/−^ mouse, pw5–17) and later stages of secondary cone degeneration, *i.e.* when most cones have died (*rd1* mice, p15–30).

As previous studies reported changes in rod- and cone-contacting second order neurons in various animal models for RP [30], [47]–[49], we analyzed the morphologies of horizontal cells, rod and cone bipolar cells in *Rho* ^−/−^ and *rd1* mice and their Cx36-deficient littermates. Reorganization is evoked by disturbed glutamatergic input from photoreceptors [47]–[49]. Consistent with earlier reports [28]–[30], [38], we found that horizontal and rod bipolar cell dendrites in both RP models reached out into the ONL, presumably searching for photoreceptor input. At later disease stages, these ectopic dendrites were retracted and horizontal cells sent numerous dendrites into the INL. As ectopic horizontal cell dendrites mostly originate from the rod-contacting axonal arborization [30], it is not surprising that we did not find differences between Cx36-expressing and Cx36-deficient mice. However, also cone-contacting ON bipolar cells showed remodeling and nearly completely lost their dendrites with disease progress (Fig. 4) [29], [30]. Rod- and cone-contacting type 3b OFF cone bipolar cells retained elaborate dendritic arbors in the OPL (Fig. 5) after retracting sprouted dendrites with progressive thinning of the ONL. Thus, although we found a reorganization of rod- and cone-contacting second order neurons similar to previous studies, we failed to detect any effect of Cx36 deficiency on retinal remodeling.

We also quantitatively assessed features directly associated with the onset and later stages of secondary cone degeneration. The loss of COS is a first indication of cone degeneration and shows a clear center-to-periphery gradient in *Rho* ^−/−^ mice [2]. A significant reduction of the COS density in the central part of the dorsal retina indicated the beginning of cone degeneration in *Rho* ^−/−^Cx36^+/+^ and *Rho* ^−/−^Cx36^−/−^ mice at pw5. Loss of COS persisted over a long period of time (>pw12, Fig. 6). However, the number of COS decreased similarly in Cx36-expressing and Cx36-deficient siblings.

The fast degenerating *rd1* mouse model was used to assess cell death in cones and loss of cone photoreceptors as late events in secondary cone degeneration. From p15 until p30, *rd1* mice lost almost 60% of their cones as shown by quantifying the number of cones per 100 µm in the OPL (Fig. 7). Consistently, a high number of TUNEL-positive cells were found between p21 and p30. Because the major phase of rod death ends around p20 [2], TUNEL-positive cells at p30 most likely represent dying cones. For both *rd1* mouse mutants, we cannot exclude that a small proportion of TUNEL-positive cells represented rods, suggesting that the number of dying cones might be slightly overestimated in both genotypes. However, if Cx36 had an influence on late stages of cone degeneration, the number of dying and lost cones, respectively, should have been reduced in Cx36-deficient *rd1* mice. This was not the case.

### Rod-cone Gap Junctions do not Mediate a Bystander Effect in RP Mouse

As we found no influence of Cx36 deletion on secondary cone degeneration, we exclude a Cx36-dependent gap junction-mediated spread of a cell death-inducing signal from dying rods to healthy cones.

Other studies, however, demonstrated that gap junctions can propagate cell death-inducing signals in cell lines [39], [40] and even retinal neurons [9], [10]. Cusato et al. (2003) reported that dying cells are clustered in the developing retina and that this clustered cell death is reduced by the gap junction inhibitor carbenoxolone [9]. The authors hypothesized that a cytotoxic byproduct of apoptotic cell death is passed to neighboring cells via gap junctions [9]. Paschon et al. (2012) used an acute trauma model of the retina to show that gap junction blockers - one of them rather specific for Cx36 - reduced the spread of apoptosis in retinal neurons after injury [10]. In contrast to these models, photoreceptor death in RP mice is not mediated by classical apoptosis but most likely involves a non-apoptotic, alternative cell death mechanism [50] characterized by a deregulation of cGMP metabolism, down-regulation of transcription factors, activity of histone deacetylases and excessive activation of calcium-dependent proteases [27], [51], [52]. Thus, we may have failed to detect a Cx36-dependent gap junction-mediated bystander effect in RP because non-apoptotic cell death in rods may not produce gap junction-permeable cytotoxic metabolites (*e.g.* IP_3_) able to induce cell death in neighboring cells.

Another reason why rod-cone coupling may not play a role in secondary cone degeneration is that gap junctions between rods and cones may close as soon as rods begin to die. Because Cx36 is not expressed in rods [12] in the mouse retina and the connexin on the rod side is not known so far, we can only speculate on the properties of the rod connexin. The conductance of most connexins is decreased by a rise in the intracellular calcium concentration and a drop in pH [44] – two common incidents during cell death [27], [53], [54]. In contrast, gap junctions exclusively made of Cx36 were reported to decrease their conductance upon intracellular alkalinization and not acidification [55]. This may represent the reason why gap junctions made of Cx36 were reported to remain open during ischemia [56], thereby mediating bystander killing. However, as the connexin on the rod side is not Cx36 but another connexin, it is conceivable that the gap junction between rods and cones is closed when rods begin to die. Additionally, intracellular signaling cascades may contribute to the closure of the rod-cone gap junction. Cx36 lowers its conductance, for example, in response to activation of the protein kinase G pathway [57], a pathway which was reported to play a role in retinal degeneration [58]. A similar mechanism may also apply to the unknown rod connexin.

Also, we cannot entirely exclude the possibility that another gap junction protein may compensate for the deletion of Cx36 as we did not directly assess rod-cone coupling in RP mouse models. However, other studies on mice [14]–[16] have shown that deletion of Cx36 is sufficient to disrupt rod-cone coupling. Thus, it seems highly unlikely that - despite the deletion of Cx36 - rod-cone coupling is functional in *Rho* ^−/−^Cx36^−/−^ and *rd1*Cx36^−/−^ mice.

Gap junctions were not only discussed to propagate cell death from dying cells to healthy neighbors but were also shown to mediate a *positive* bystander effect [59]–[61]. For the mouse, Striedinger et al. (2005) reported that Cx36 is upregulated in response to retinal lesioning. Blockade of gap junctions with carbenoxolone resulted in an increased extent of secondary cell loss in this mouse model. Thus, Cx36 may also protect neighboring cells from cell death after traumatic injury of the retina [62]. If Cx36-containing rod-cone gap junctions mediated a positive bystander effect, deletion of Cx36 would have led to an acceleration of secondary cone degeneration in RP mouse models. However, the time course and extent of cone death were unchanged in Cx36-deficient mice, ruling out a negative and positive bystander effect for Cx36-dependent gap junctions.

In summary, our study provides the first conclusive evidence that a Cx36-dependent gap junction-mediated bystander effect, postulated by Ripps [8], is not involved in secondary cone degeneration in mouse models for RP as the deletion of Cx36 on the cone side of the rod-cone gap junction had no effect on the secondary death of genetically healthy cones. However, since the gap junction-mediated transfer of death signals is not the only possible mechanism to mediate bystander killing, we cannot exclude that extracellular propagation of toxic intermediates [39] may contribute to cone degeneration in RP.

## Materials and Methods

Unless stated otherwise, all chemicals were purchased from Carl Roth GmbH (Karlsruhe, Germany).

### Ethics Statement

All experiments were carried out in accordance with the institutional guidelines for animal welfare of the University of Oldenburg, following the standards described by the German animal protection law (*Tierschutzgesetz*). The mere killing of mice for tissue analysis is registered with the local authorities (*Niedersächsisches Landesamt für Verbraucherschutz und Lebensmittelsicherheit*) and reported on a regular basis as demanded by law but needs no further approval if no other treatment is applied before killing.

### Transgenic Animals

Photoreceptor degeneration was studied in *Rho* ^−/−^ mice [17] and in *rd1* mice (Charles River, Wilmington, MA) [63]. Both RP models were crossbred with Cx36^−/−^ mice (C57Bl6/N genetic background) [23], resulting in *Rho* ^−/−^ and *rd1* mice with a heterozygous deletion of Cx36. The offspring generation of *Rho* ^−/−^Cx36^+/−^ and *rd1*Cx36^+/−^ mice was intercrossed to obtain homozygous Cx36-expressing and Cx36-deficent littermates for both RP mouse models. Mice were genotyped for alterations in genes encoding for *rhodopsin, rod cGMP phosphodiesterase subunit beta* (*Pde6β*) and *Cx36* by polymerase chain reaction analysis of tail DNAs using sets of primers listed in Table 1. To minimize differences in genetic background, comparative analyses were performed on littermates. For *Rho* ^−/−^Cx36^+/+^ and *Rho* ^−/−^Cx36^−/−^ mice, the ages of 5, 9, 12 and 17 weeks were analyzed. *Rd1*Cx36^+/+^ and *rd1*Cx36^−/−^ mice were analyzed 15, 21, and 30 days after birth, respectively. Additional control experiments were performed with age-matched wt animals corresponding to the respective genetic backgrounds of the transgenic mouse strains used (*Rho* ^−/−^: C57Bl6/N, Charles River; *rd1*: C3A.BLiA-*Pde6b*+/J, The Jackson Laboratory, Bar Harbor, ME).

**Table 1:** Primers used for mouse genotyping.

Primer	Primer sequence 5′ to 3′	References*
RhoF	TCT CTC ATG AGC AGC CTA AAG	[17]
RhoR	ATG CCT GGA ACC AAT CCG AG	[17]
Rho KO	TTC AAG CCC AAG CTT TCG CG	[17]
Rd1 2.1F	TGT TGC TCT GCG GTA AGA TG	[66]
Rd1 2.1R	TCC CTC AGT CTG GGA TCA AT	[66]
RD3	TGA CAA TTA CTC CTT TTC CCT CAG TCT G	[66]
RD4	GTA AAC AGC AAG AGG CTT TAT TGG GAA C	[66]
HRT EPS7	CAG TAA ATC GTT GTC AAC AGT TCC	[23]
EX3T Intron	CTG TTC AAG GAC TGG TAA GCG CTG	[23]
EX36 DSP4	GTC TCC TTA CTG GTG GTC TCT GTG	[23]

* related to primer sequences and/or PCR conditions used.

### Retinal Tissue Preparation and Immunohistochemistry

Animals were anesthetized with CO_2_ and killed by cervical dislocation. Eyes were enucleated and prepared in Ringeŕs solution containing (mM) 137 NaCl, 5.4 KCl, 1.8 CaCl_2_, 1 MgCl_2_, 10 D-glucose, and 5 HEPES, pH 7.4. Cornea, lens and vitreous body were removed from the eyecup. Eyecups for vertical retina sections were fixed either for 20 minutes in 2% paraformaldehyde (PFA; Riedel de Haen, Seelze, Germany) or for 60 minutes in 4% PFA in phosphate-buffered saline, pH 7.4 (PBS), followed by several washing steps in PBS. Eyecups were cryoprotected in sucrose-containing PBS solutions (10%, 20%, 30% sucrose) and embedded in cryoblock (Medite GmbH, Burgdorf, Germany) at −20°C. Vertical sections (18–20 µm for morphological analysis; 12 µm for quantitative analysis) were cut on a cryostat (Bright, Huntingdon, United Kingdom). Sections from littermates and same-aged wild-type mice were collected on the same slide to ensure equal treatments. Cryosections were rinsed either in 0.1 M phosphate buffer (PB, pH 7.4) or in Tris-buffered saline (TBS, pH 7.4) containing 0.3% Triton X-100 (TBST) and blocked either with 10% normal goat serum (Sigma-Aldrich Chemie GmbH, Steinheim, Germany) or 10% normal donkey serum (Dianova GmbH, Hamburg, Germany) in TBST. Primary antibodies were applied overnight at 4°C. After several washing steps, slices were incubated with secondary antibodies for at least two hours. Finally, sections were rinsed again and subsequently mounted in Vectashield (Vector Laboratories, Burlingame, CA). Primary (Table 2) and secondary antibodies were diluted in blocking solution. Secondary antibodies were conjugated to Alexa Fluor 488 or 568 (Invitrogen, Karlsruhe, Germany) or Cy3 (Jackson Immunoresearch, West Grove, PA). Experiments, in which primary antibodies were omitted, were performed to control for non-specific binding of secondary antibodies. In some experiments, nuclei were stained by adding the nucleic acid stain TO-PRO-3 (Invitrogen) to the secondary antibody solution (dilution 1∶1,000) or by using Vectashield with DAPI (Vector Laboratories).

**Table 2:** Primary antibodies used in this study.

	Immunogen	Species, type, dilution	Source (Cat. No.)
Calbindin D-28K	Calbindin D-28K, chicken, full-length amino acid sequence	Mouse, monoclonal, 1∶5,000	Swant, Marly, Switzerland (300)
Calbindin D-28K	Recombinant rat calbindin, D-28K	Rabbit, polyclonal, 1∶1,000	Swant, Marly, Switzerland (CB-38a)
Cone arrestin	Synthetic linear peptide, derived from aminoacid 369–389 of cone arrestin	Rabbit, polyclonal, 1∶1,000	Millipore, Billerica, MA (AB15282)
Connexin36	C-terminal region of rat and mouse Cx36,derived from amino acid 286–303	Rabbit, polyclonal, 1∶500	Invitrogen Carlsbad, CA (51–6300)
Glycogen phosphorylase	Guinea pig-anti glycogen phosphorylase	Guinea pig, polyclonal, 1∶1,000	B. Pfeiffer-Guglielmi University of Tübingen, Tübingen, Germany
G_0α_	Bovine brain G_0α_	Mouse, monoclonal,1∶500	Millipore, Billerica, MA (AB 144P)
PKARIIβ	Human recombinant protein kinase A, regulatory subunit IIβ derived from amino acids 1–418	Mouse, monoclonal, 1∶2,000	BD Bioscience, San José, CA (610625)
PKCα	Raised against a peptide mapping at theC-terminus of PKCα of human origin	Goat, polyclonal, 1∶500	Santa Cruz Biotechnology, Santa Cruz, CA (Sc-208-G)
OPN1SW (N-20)	Raised against an epitope mapping at theN-terminus of the human S-opsin proteinencoded by *OPN1SW*	Goat, polyclonal, 1∶500	Santa Cruz Biotechnology, Santa Cruz, CA (Sc-14363)
Opsin, red/green	Recombinant human red/green opsin targetingthe last 38 amino acids from theC-terminus	Rabbit, polyclonal,1∶500	Millipore, Billerica, MA (AB 5405)
Velis-3	Synthetic peptide derived from the C-terminus of rat velis-3 (MALS-3) protein	Rabbit, polyclonal,1∶1,000	Invitrogen Carlsbad, CA (51–5600)

Quantification of COS was carried out on flat-mounted retinas. Eyecup preparation was performed as described above; the dorsal orientation of each retina was marked by incision. Isolated retinae were flat-mounted on slides, fixed for 40 minutes with 2% PFA, transferred onto a filter paper (Millipore, Billerica, MA) with the photoreceptor layer up and kept in 0.1 M PB. Retinas of littermates and age-matched wild-type controls were incubated in the same well of a 6-well plate. Whole-mounts were washed in 0.1 M PB and blocked with 5% ChemiBLOCKER (Millipore) containing 0.5% Triton X-100 and 0.05% NaN_3_ for one hour. Primary antibodies were applied for 5 days at 4°C (Table 2). Secondary antibodies were applied overnight at 4°C. After several washing steps whole-mounts were mounted in Vectashield.

### Terminal Deoxynucleotidyl Transferase dUTP Nick end Labeling (TUNEL Assay)

To determine the number of dying cells, a TUNEL assay was performed on vertical cryostat sections using an *in situ* cell death detection kit (Fluorescein or TMR; Roche Diagnostics GmbH, Mannheim, Germany) in accordance with the manufactureŕs instructions.

### Fluorescent Image Acquisition

Images for morphological comparisons between transgenic animals and for the quantification of COS were taken with a Leica TCS SL confocal microscope, as described previously [64]. Scanning for morphological comparisons was performed either with a 40×/1.25 oil plan apochromat or with a 63×/1.32 oil plan apochromat objective (z-axis step size 0.2 µm). Confocal images for quantification of cone outer segments (COS) were taken either with a 20×/0.5 plan fluotar objective or a 40×/0.7 plan fluotar objective (z-axis step size 0.5–0.8 µm). Unless stated otherwise, images are presented as maximum projections of z-stacks of 2–3 µm thickness. To quantify photoreceptors and TUNEL-positive cells, images were taken using an Axio Imager Z1 ApoTome microscope, equipped with a Zeiss Axiocam digital camera and Zeiss Axiovision 4.7 software. Complete vertical sections were scanned using the Mosaix mode of Axiovision 4.7 at 20× or 40× magnification. Images were superimposed and slightly adjusted for brightness and contrast in Photoshop CS4 (Adobe, San Jose, CA).

### Quantification and Statistical Analysis

Retinas of each experimental group were processed under identical conditions with respect to tissue dissection, incubation steps and microscopic evaluation. Quantification of cone photoreceptors and TUNEL-positive cells was performed as previously described [65]. For the quantitative COS analysis, confocal maximum projections (thickness 3–5 µm) of the outer segment region, ranging from the optic nerve up to the dorsal and ventral edge of the retina, were intensity adjusted and assembled to one montage, covering the dorsal-ventral axis of the whole-mounted retinas, in Photoshop CS4. For each retina, ROI were defined at 50% and 75% dorsal and ventral of the optic nerve, respectively. The density of COS was manually determined in an area of 100×200 µm^2^. Cone photoreceptor and TUNEL quantification was carried out only in the central retina, which was defined as the area ranging from the optic nerve up to a distance of 1,000 µm towards the retinal periphery. Slices for quantification were taken in the immediate vicinity of the optic nerve. The summarized results display the manually determined number of cones and TUNEL-positive cells per 100 µm retina length.

For statistical comparisons within one genotype and between genotypes the unpaired, two-tailed Students t-test was used. Quantitative data represent the means of at least three different littermates and appropriate wild-type control mice per genotype and age. Error bars in the figures indicate standard deviation. Levels of significance are indicated as follows: *, p<0.05; **, p<0.01; ***, p<0.001.
